# Acute Pain in the African Prehospital Setting: A Scoping Review

**DOI:** 10.1155/2019/2304507

**Published:** 2019-04-16

**Authors:** Andrit Lourens, Michael McCaul, Romy Parker, Peter Hodkinson

**Affiliations:** ^1^Division of Emergency Medicine, University of Cape Town (UCT), Cape Town, South Africa; ^2^Division of Epidemiology and Biostatistics, University of Stellenbosch (SU), Cape Town, South Africa; ^3^Department of Anaesthesia and Perioperative Medicine, University of Cape Town (UCT), Cape Town, South Africa

## Abstract

**Background:**

Acute pain is a common reason for seeking prehospital emergency care. Regrettably, acute pain is often underestimated and poorly managed in this setting. The scoping review was conducted to gain insight into existing research on the topic and to make recommendations for future work.

**Objectives:**

To identify all available evidence related to acute pain assessment and management in the African prehospital setting, describe the extent of the evidence, encapsulate findings, and identify research gaps.

**Methods:**

The scoping review considered primary and secondary research related to acute pain assessment and management of both medical and traumatic origins in all age groups in the African prehospital setting. The search strategy aimed to identify published, unpublished, and ongoing research which met the inclusion criteria. Potentially eligible studies were identified by a comprehensive search of electronic databases, trial registers, dissertation/thesis databases, grey literature databases, and conference proceedings. Screening and data extraction were conducted independently and in duplicate.

**Results:**

The comprehensive search identified 3823 potential studies, duplicate titles were removed, and 3358 titles/abstracts were screened. Full text of 66 potentially eligible titles was screened, 60 were excluded, and six publications met the inclusion criteria. Despite recommendations for pain assessment during general patient care, most studies reported no/limited pain assessment. In general, pain management was concluded to be insufficient and not conforming to best practice.

**Conclusions:**

Only six publications addressing prehospital acute pain care in Africa could be identified, possibly indicative of a knowledge gap. Future research is indicated to enable a better understanding of the epidemiology of acute pain and barriers and enablers of acute pain care and to develop evidence-based clinical practice guidelines (CPGs) catering for all EMS systems in Africa. Additionally, educational initiatives should be implemented to improve the quality of acute pain care and to monitor quality through continuous quality improvement (CQI) programs.

## 1. Introduction

Acute pain (on its own or along with other complaints) is a common reason for seeking emergency care (EC), in the prehospital and hospital emergency department (ED) setting. The prevalence of ED visits secondary to acute pain is between 38% and 91% [1–6] with prevalence in the prehospital setting, reported to range between 34% and 75% [7–14]. Acute pain is fundamentally a protective mechanism and fosters survival.

Being a stressor, acute pain activates various body systems with the potential to result in numerous physiological and psychological adverse effects. If unalleviated, acute pain is associated with worse patient outcomes [15–18], thus making pain assessment and management in the acute setting an essential aspect of quality care [18]. In addition, when considering the ethical and human rights concerns related to acute pain [17, 18], all healthcare providers (HCPs) should view it as a priority with the aim to alleviate suffering and minimising the coinciding adverse effects. Despite the high prevalence of pain in the acute setting and the associated negative effects, research highlights the poor and often insufficient assessment and management of acute pain [1, 8, 10, 15, 19, 20]. Three main barriers have been identified as contributing factors to poor prehospital acute pain management, namely, provider perceptions and beliefs, patient-related barriers, and system barriers [21].

Adequate pain management in the prehospital setting is both realistic and achievable, but improvement will require an understanding of the aforementioned pain management barriers and limitations in emergency medical services (EMS) systems, development of pain management policies/strategies [21], and investment in pain management education [18, 21]. French et al. [22] demonstrated that, after a 3-hour educational intervention, paramedics exhibited an increased understanding of the principles of pain and pain management with practitioners subsequently more likely to document the outcomes of interventions and the delivery of nonpharmacological pain management. A follow-up study six years later showed those practitioners' knowledge and perception of pain, and pain management remained improved [23].

Knowledge and perceptions about pain should include the understanding that pain is influenced by various factors like culture, gender, age, language, context, previous experiences, level of consciousness, and cognition. [17]. Pain assessment and management are prejudiced by HCPs' beliefs, attitudes, and opinions of pain with studies reporting that HCPs generally underestimate pain [15, 18, 24–27] with the underestimation increasing with practitioner experience [27]. Prehospital education and levels of qualifications differ significantly from country to country, with emergency care providers (ECPs) worldwide practising according to different protocols or guidelines, scope of practice, and standard operating procedures. Levels of qualifications may vary from basic life support (BLS) practitioners with a limited scope of practice and skill set aimed at assuring basic vital functions through to advanced life support (ALS) practitioners with a broader scope of practice including more invasive skills and medications. In Africa, access to EC in the prehospital setting is very limited; nevertheless, this is a rapidly developing area as healthcare systems evolve and countries aspire to establish/develop EMS systems [28].

Although globally, pain and pain management are well-researched topics, given the diversity of EMS systems in Africa and the role of culture, gender, attitudes, and beliefs about pain, acute pain assessment, and management in the African prehospital setting are a pertinent area deserving in-depth exploration [28–30]. Furthermore, given that the literature reports that acute pain in the African in-hospital setting is highly prevalent and poorly managed, it is likely that acute pain in the prehospital setting is also a challenge in Africa [24, 31, 32]. In low-resource settings, like most African countries, various factors have been identified which may hinder effective pain management. These include insufficient education and training of HCPs, lack of resources, and opioid analgesics and malalignment of government priorities and policies [33].

The methodology behind scoping reviews allows for evaluating a broad research question with the intent to summerise research findings and to articulate what is known about a specific topic [34–38]. This review will provide insight into existing prehospital acute pain assessment and management practice and research in Africa to clinicians and policymakers and allow for making recommendations to the profession as a whole and specifically to researchers through (1) identifying and mapping the range and nature of evidence; (2) identifying research gaps in the existing literature; (3) summarising research findings; and (4) informing future research, related to acute pain assessment and management in the prehospital setting in Africa.

## 2. Methods

Inclusion and exclusion criteria were considered in terms of type of participants, concept, context, and sources.

### 2.1. Inclusion/Exclusion Criteria

#### 2.1.1. Type of Participants

The scoping review considered research in any age group, with patients managed by ECPs, physicians, and/or nurses in the prehospital setting, in Africa. Studies relating to neonates were excluded as it is beyond the scope of the review.

#### 2.1.2. Concept

The concept of interest was the assessment and management of acute pain of both traumatic and medical aetiology in the African prehospital setting.

#### 2.1.3. Context

The context of the scoping review was the prehospital setting and only considered research conducted on the African continent. Prehospital refers specifically to care provided before or during transportation of the patient to hospital by EMS, and consequently, studies conducted in the aeromedical (helicopter and fixed-wing) setting and ground ambulance services were eligible. Studies related to interfacility transfers of critically ill and injured patients were excluded as pain assessment and management may be influenced by prior treatment and therefore should probably not be compared to pain care in the primary setting.

#### 2.1.4. Type of Sources

The research designs considered for inclusion were primary research designs [experimental designs (randomised controlled trials and nonrandomised controlled trials), observational designs (cohort studies, case-control studies, cross-sectional studies and surveys) and qualitative designs] and secondary research designs (systematic reviews and meta-analysis and evidence-based CPGs), whereas case reports, case series, and literature reviews were excluded.

### 2.2. Search Strategy

The search strategy aimed to identify published, unpublished, and ongoing research. Potentially eligible studies were identified by comprehensively searching the following electronic databases up to December 2018: MEDLINE, Science Direct, Scopus, Google Scholar, EBSCOhost (Academic Search Premier, Africa Wide Information, CINHAL and Health Source: Nursing/Academic Edition), the Cochrane Central Register for Controlled Trials (CENTRAL), Web of Science (all databases), African Journals Online (AJOL), and Sabinet African Journals (African Journal Archive) (Supplementary Appendix 1). The International Guidelines Library and National Institute for Health and Care Excellence (NICE) were searched for CPGs. Searches were limited by year of publication (from 1 January 2000) but not by language.

The ClinicalTrials.gov register and World Health Organization (WHO) International Clinical Trials Registry Platform were searched to identify relevant protocols, ongoing studies, and unpublished studies up to 29 November 2018. The ProQuest Dissertations and Theses database and Sabinet WorldCat Dissertations were searched for potentially relevant dissertations and theses (search up to December 2018). The grey literature database was searched for potentially relevant grey literature, and the ERIC ProQuest database was searched for potentially relevant conference abstracts or proceedings. A further effort was made in August 2018 to find grey literature by contacting emergency medicine (EM) leaders and EM societies in the African region as well as searching the following databases: OpenThesis, Networked Digital Library Theses and Dissertations, Agency for Healthcare Research and Quality, WHO: Global Index Medicus, OpenUCT, Scopus (conference proceedings), and Database of African Theses and Dissertation including Research (DATAD-R). The reference lists of included studies/thesis were reviewed for eligible publications. The corresponding authors of included studies were contacted to identify additional relevant studies (published, unpublished, or ongoing).

### 2.3. Selecting Eligible Studies

Search results were imported to the Covidence online software [39]. A two-stage process was utilised to identify eligible studies. In stage one, two reviewers (AL and MM) independently and in duplicate reviewed the search results for potentially eligible studies (titles/abstracts) using the prespecified inclusion/exclusion criteria. After concluding screening, the full-text reports of potentially relevant titles/abstracts were retrieved for final eligibility (Supplementary Appendix 2) assessment (stage two) by the two reviewers, independent and in duplicate. Disagreements were resolved through discussion and wherever necessary mediated by a third party (RP).

### 2.4. Data Extraction

Data of the included studies were captured independently and in duplicate by two reviewers (AL and MM) on a data extraction form (Supplementary Appendix 3). The following information was recorded: author/s, year of publication, publication type (journal article, dissertation, conference proceedings, etc), study aim/s, study design, study location (city and country), study setting, data collection method (interviews, questionnaires, patient care report (PCR) reviews, etc.), sampling strategy and sample size, type of participants [(adult or paediatric) and (trauma or medical)], medication information (class of medication, medication administered, dose administered, repeated dosages, and/or rescue analgesia), type of pain assessment, route of administration [inhaled, oral, intranasal (IN), intramuscular (IM), and intravenous (IV)], nonpharmacological management, and main results. Disagreements were resolved through discussion.

## 3. Results

### 3.1. Search Results

**Figure 1:**
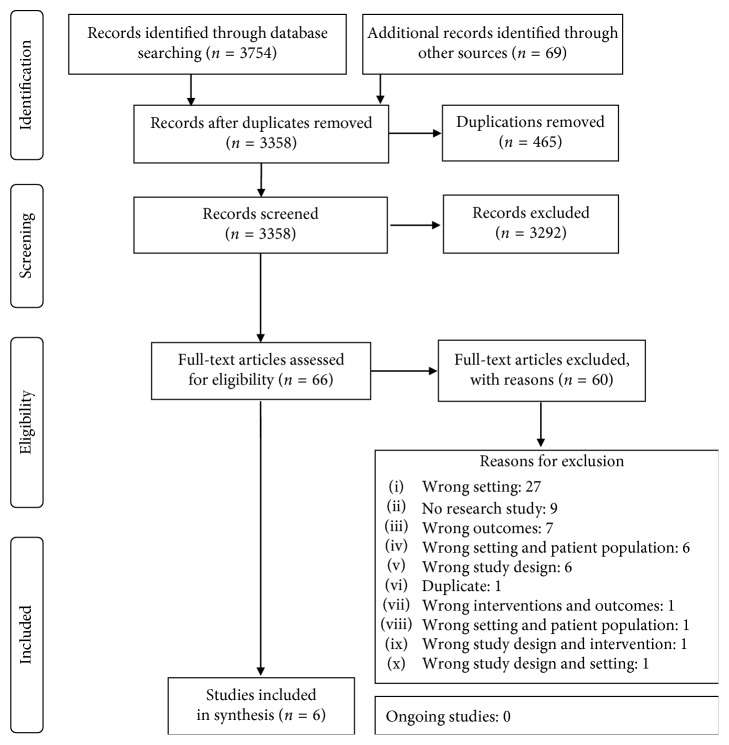
Flow diagram of the study selection.

The comprehensive search identified 3823 potential studies. Duplicate titles (465) were removed, after which 3358 titles/abstracts were screened. Sixty-six titles/abstracts were potentially eligible with 3292 records excluded. The full-text articles of the 66 potentially eligible titles were retrieved, and eligibility criteria were applied. Sixty articles/publications were excluded and six included (Figure 1) in the scoping review.

### 3.2. Characteristics of Included Studies

Of the six included titles, four were peer-reviewed journal articles and one was a thesis dissertation. The sixth was grey literature published by the Professional Board for Emergency Care (PBEC), Health Professions Council of South Africa (HPCSA), and obtained through the authors' knowledge of the field of EC. One study utilised a mixed methods approach, three were observational descriptive research, one was an interrupted time series analysis, and the remaining were evidence-based CPGs. All six studies were written in English and published between 2012 and 2018. Five originated from South Africa (SA) and one from Rwanda (Table 1).

### 3.3. Key Results/Findings of Included Studies

The key features of the six included papers are synthesised in Table 2.

#### 3.3.1. Acute Pain Prevalence

None of the included papers reported acute pain prevalence in the African prehospital setting. Nevertheless, in Phase 1 (quantitative phase) of the mixed methods study by Mulder [40], respondents (*n*=57) to the survey indicated that 2% encountered <1 patient requiring analgesia per month, 28% encountered between 1 and 5 patients, 36% between 5 and 10, 19% between 10 and 15, 6% between 15 and 20, and 9% more than 20 patients per month.

#### 3.3.2. Aetiology of Acute Pain

In the review of PCRs (*n*=530), Matthews et al. [41] found the following causes for initiating analgesia: soft tissue injuries including burns (*n*=74, 14%, 95% CI: 11–17), fracture, amputations or dislocations (*n*=132, 25%, 95% CI: 21–29), stabbing or gunshot wounds (*n*=52, 10%, 95% CI: 7–13), chest pain (*n*=226, 42%, 95% CI: 38–47), and nontraumatic pain including back pain (*n*=42, 8%, 95% CI: 6–11). In four cases, diagnostic notes were not recorded. Participants (*n*=60) in the study by Vincent-Lambert and De Kock [42] identified fractures (100%), dislocations (96.7%), burns (95%), chest pain (90%), and severe soft tissue injuries (81.7%) as conditions commonly associated with noteworthy pain and the need for analgesia.

#### 3.3.3. Pain Assessment (Initial and Reassessment)

Generally, pain assessment practice in the included studies was poor. Matthews et al. [41] reported that the numeric rating scale (NRS) assessment was recorded in 111 (21%, 95% CI 18–25) cases, whereas a second NRS assessment was recorded in only 34 (6%, 95% CI 4–9) cases. In the descriptive cross-sectional study by Cox et al. [43], none of the 353 paediatric burns victims had their pain management assessed using a pain scale prior to admission to the burns unit.

In phase 1 of the study by Mulder [40], respondents indicated that, to initiate analgesia, a comprehensive picture is required and decisions are not based on a single isolated factor. Additionally, respondents (81%) reported that both a decrease in pain score and physiological changes are indications to stop pain management. In the second phase (quantitative), the patient's expression of pain was identified as the main determinant in the decision to initiate analgesia. Despite the questionnaire indicating that practitioners incorporated pain scores during pain management, most of the interviewees (*n*=5) expressed that a pain score is not a good indicator for initiating analgesia. The patient's expression of comfort was deemed a good indicator for stopping analgesia, whereas the practitioner's opinion of the patient's pain in terms of the patient appearing comfortable and the patient requesting the practitioner to stop pain management was identified as factors contributing to the cessation of analgesia. Vincent-Lambert and De Kock [42] stated that participants used verbalised pain relief, decreased pain score, and decreased heart rate as perceived end-points of analgesia (effective pain relief).

The evidence-based CPGs for the South African prehospital setting by the Health Professions Council of South Africa (HPCSA) [44] recommend the following conditions in terms of pain assessment. The description partly reproduces the wording as captured in the HPCSA CPGs [44]:

- Use age-appropriate pain scales as part of general patient care
- All trauma patients should be considered candidates for pain relief
- In labour, meet the mother's pain relief expectations
- All patients who received analgesia must be reassessed every 5 minutes (using age-appropriate pain scale)
- Observe patients for evidence of severe adverse effects like sedation, hypotension, hypoxia, and anaphylaxis
- Presence of severe adverse effects demonstrates the need to stop further administration
- EC courses should teach nationally standardised age-appropriate pain scales

#### 3.3.4. Factors Influencing Decision-Making

Respondents to the study by Vincent-Lambert and De Kock [42] indicated that the following factors were considered during the decision-making of whether to administer morphine for analgesia: level of pain being experienced, patient's desire for pain relief, practitioners' fears of adverse effects and transportation (mode, time, and conditions). During decision-making, interviewees in the second phase of the study by Mulder [40] reported mechanism of injury, the need to move the patient, factors causing emotional influences like socioeconomic status, insurance status, age, gender, and the practitioner perceiving the injury to be painful based on personal experience or looking at the injury as contributing factors. Physiological indicators, influenced by external stimuli particularly in the prehospital setting, were deemed a poor reference for decision-making unless the patient was intoxicated or altered.

#### 3.3.5. Nonpharmacological Management of Acute Pain

Limited results related to the nonpharmacological management of acute pain were obtained from the included papers. For paediatric burns victims, a Burnshield® dressing was applied by EMS in 22 (6.2%) children and 251 (71.1%) children at community health centres [43]. HPCSA [46] CPGs recommends cooling and covering burns and the immobilisation of fractures. Scott et al. [45] found that, after the implementation of the CQI program, there was a significant improvement in the percentage of extremity fractures splinted (pre-CQI: 87.5% (*n*=335) vs post-CQI: 92.6% (*n*=393); *p*=0.019).

#### 3.3.6. Pharmacological Management of Acute Pain

The main pharmacological pain management recommendation of evidence-based CPGs by the HPCSA [44] is shown in Table 3. The description in Table 3 partly reproduces the wording as captured in the HPCSA CPGs [44].

For paediatric burns victims, parents and medical staff used paracetamol most frequently, whereas IV morphine in combination with oral paracetamol was administered if transported to burns units by ambulance [40]. As evident from PCRs, Matthews et al. [41] reported the following analgesia practices by ALS practitioners. Morphine with a median dose of 4 mg (IQR 3–6) was administered in 371 (70%, 95% CI: 66–74) cases and a total of ≥5 mg morphine administered in 278 (75%, 95% CI: 70–79) cases. One dose of morphine was administered in 268 (72.2%, 95% CI: 67–77), two doses in 86 (23%, 95% CI: 19–28), and three doses in 18 (5%, 95% CI: 3–8) cases. Coadministration of morphine with nitrates occurred in 47 (24%, 95% CI: 18–30) cases and morphine with ketamine in three (33%, 95% CI: 7–70) cases. Sublingual nitrates were administered in 197 (37%, 95% CI 33–41) cases and ketamine in nine (1.7%, 95% CI 1–3) cases [41].

Fifty-one participants (85%) in the internet-based survey by Vincent-Lambert and De Kock [42] indicated a preference for a high-dose morphine regimen (0.1 mg/kg followed by 0.05 mg/kg after 5 minutes), whereas nine participants selected a low-dose morphine regimen (0.05 mg/kg followed by 0.025 mg/kg after 5 minutes) in hemodynamically stable patients with severe pain. The most common reasons for low-dose regimen were concerns for nausea/vomiting, hypotension, respiratory depression, blunting diagnostic procedures at the ED, dose enough to dull pain to a tolerable level, and the belief that patients sometimes lie about the extent of their pain. The rationale for selecting the high-dose regimen was based on the following opinions of participants: adverse effects are more depended on the rate of medication administration than the dose; aim of relieving pain instead of merely blunting pain; pain may be harmful to patient outcomes; and if a definite pain response is present, patients will not experience adverse effects.

Scott et al. [45] found that after the implementation of the CQI program, there was a significant improvement in the administration of pain control (pre-CQI: 85.1% (*n*=335) vs post-CQI: 93.6% (*n*=393); *p* < 0.001) in trauma patients.

Morphine was specified as the method of analgesia in 68% of respondents in Phase 1 of Mulder's [40] study. For practitioners with ketamine and morphine in their scope of practice, ketamine was preferred in terms of onset of action and efficacy in trauma by interviewees (Phase 2). Some practitioners deemed a combination of ketamine and morphine more effective. In the absence of immediate life-threatening conditions, interviewees indicated that pain management takes the highest priority and that without pain management further management may not be possible.

#### 3.3.7. Study Conclusions

Cox et al. [43] determined that health staff were unfamiliar with provincial burns guidelines and analgesic drug dosages; hence, the study identified pain management as one of the six major shortfalls in the implementation of provincial burns guidelines (Western Cape, SA). Matthews et al. [41] concluded that, in the study setting (SA), prehospital pain management is likely haphazard, ineffective, and not conforming to current best practice. Furthermore, morphine is administered at low dosages, and there was limited evidence of pain assessment using a pain scale. Multimodal pain management in the prehospital setting is restricted probably due to the limited availability of alternative medications. Finally, the study urged for continuous pain care education and the development of prehospital pain management CPGs. Much like Matthews et al. [41], Vincent-Lambert and De Kock [42] recommend the development of pain management protocols for the SA prehospital setting and found that pain assessment using a pain score is lacking. Nevertheless, SA ALS practitioners seem to consider various vital factors during pain management decision-making. Further, the authors were concerned with the practice of administering the morphine loading dose in a measured approach likely resulting in a delayed onset or failure of pain relief. The study by Scott et al. [45] demonstrated that the CQI programme significantly improved both the pharmacological and nonpharmacological management of pain and concluded that the CQI programme led to an immediate improvement in prehospital care delivered as well as an improvement over time. Mulder [40] concluded that the approach to pain management of SA ALS practitioners indicates a dynamic thought process. Internal factors such as previous experience, personal perceptions, and opinions and external inputs like the patients' perception, pain score, physiological indicators, the mechanism of the injury, and the required interventions are factors influencing clinical decision-making in terms of acute traumatic pain management.

## 4. Discussion

From the results of the scoping review, it is evident that high-quality research into prehospital acute pain assessment and management in Africa is significantly lacking. Despite extensive searches, only six papers addressing the topic could be identified. Furthermore, although Cox et al. [43] met the eligibility criteria for inclusion in the scoping review, the study provided very limited information and insight into the assessment and management of acute pain associated with burn injuries in the prehospital setting. In comparison with the volume and range of prehospital pain research conducted in high-income regions like North America, Australasia, Europe, and the United Kingdom (UK), the shortfall in this field in Africa is irrefutable [7–15, 18–23, 25, 26].

### 4.1. EMS Systems and Research in Africa

Both the WHO [46] and the World Bank [47] declared a decade ago that EMS is a fundamental part of the national health systems of low-income and middle-income countries, and that governments and ministries of health of these countries should pay attention to and promote the development of EMS systems as well as prioritise investment. Due to the knowledge gap related to EMS systems in low- and middle-income countries, research should aim to determine the necessity for EMS systems, develop a better understanding of the conditions/diseases which may be addressed by or benefit from well-established EMS systems (for example, time-sensitive conditions like acute coronary syndrome and severe trauma), and examine possible solutions to region-specific problems [46, 48]. Furthermore, because EC is a neglected research area, development of and defining research priorities are problematic [49] but necessary to focus and direct prehospital research.

Access to EMS in most low- and middle-income countries including the African continent is very limited [49–52]. According to Mould-Millman et al. [28], 61.1% of African countries have no evidence of EMS systems. Less than 9% of Africans have access to an EMS system, with injury (commonly associated with acute pain) being the leading reason for EMS transportation. Forty-eight percent of systems utilised laypersons trained in first aid (tier-one) as responders and 96% medically trained (tier-two) responders of which 84% were BLS practitioners. In terms of appropriate pain management, what is of concern is that first aid-trained and BLS practitioners will predominantly manage pain with nonpharmacological methods only and to a lesser degree with pharmacological methods, which will be limited to medications such as inhaled nitrous oxide (Namibia and South Africa) [45, 53], other inhaled analgesics, like penthroxyflurane (SA) [45], or oral analgesics like paracetamol (Ghana) [54].

Despite literature describing the necessity for and importance of research for the development of EMS systems in Africa [47, 49], research in Africa and particularly in the prehospital setting remains challenging in terms of research funding, research frameworks and governance, research capacity, and clear research priorities. Worldwide, the majority of research is conducted in high-income countries with the Global Forum on Health Research [55] stating that the 10–90 gap, whereby <10% of health research funding is allocated to research in developing countries where more than 90% of preventable health issues occur, persists. As described above, some of these preventable health issues and the incurring burden may benefit from or be addressed by quality EMS systems [47, 48]. The assessment of national health research systems (NHRS) in the WHO Africa region in 2015 found that when compared to the 2003 and 2009 NHRS assessments, some countries in the African region had made advancements in developing certain functions of their NHRS. However, other countries in the region remained without NHRS [56]. To establish prehospital research principles for Africa, Mould-Millman et al. [48] recommend including, among others, the development of methods to accurately gather data related to emergency conditions (commonly associated with pain) in Africa, to measure the efficacy of basic prehospital EC (pain care is an essential part of EC), to develop region-specific prehospital research priorities, and to align these priorities with the global research agenda. To address the lack of research capacity, the focus should be placed on education and training to conduct quality and meaningful research [48].

Considering the limited number and methodological quality of the included research, this scoping review exposes the paucity of high-quality prehospital acute pain research in Africa. Except for the evidence-based CPG, no high-level evidence in the form of RCTs or systematic reviews and meta-analysis examining pain interventions in African prehospital setting could be identified. Furthermore, it is noteworthy that none of the guidelines adapted, adopted, or contextualised for the purposes of the CPGs [44] originated from Africa. Additionally, the studies contained no or very limited epidemiological data, making describing acute pain and developing an understanding of the extent of the acute pain burden in the African prehospital setting problematic. It is desirable to develop a broader understanding of how ECPs' knowledge, opinions, and behaviours influence pain care in the form of qualitative research as well as how CQI projects may improve acute pain care in the African prehospital setting.

### 4.2. Acute Pain Prevalence in the Prehospital Setting

As mentioned, none of the studies included in the scoping review investigated or reported the prevalence or any other noteworthy epidemiological characteristics of acute pain in the African prehospital setting. As previously stated, international studies indicate that acute pain in the prehospital setting is prevalent and often undertreated [7–11, 13, 15]. If one merely considers the high trauma rate in the African region, it is reasonable to anticipant that acute pain in the African prehospital setting will be prevalent. Because pain management is a human right, for all citizens of the world [57, 58], and its presence brings about unnecessary suffering, it must be emphasised, scrutinised, and addressed.

In comparison to communicable diseases (like malaria, TB, and HIV/AIDS), primary healthcare (child immunization), and basic resources like running water, pain management would seem to be a low priority in the health systems of low- and middle-income countries, with a paucity of comprehensive data on pain and pain management [58–60].

### 4.3. Acute Pain Assessment in the Prehospital Setting

Continuous assessment of the severity of acute pain forms an integral part of acute pain management as it provides the basis for decision-making [15, 61]; nevertheless, barriers are numerous. The subjective nature of pain, cultural, religious, and personal beliefs of patients, language barriers, lack of education and knowledge (practitioners and patients), attitudes, and practices on the part of HCPs all make pain assessment a challenge [15, 61–63]. Three of the studies [40–42] included in the review discussed and raised concerns related to acute pain assessment as practitioners did not conform with best practice which requires the use of an age-appropriate pain scale with regular reassessment [17, 44, 64, 65]. For a pain assessment tool to be applicable and suitable for prehospital use, it must be quick, not require equipment to record, be reproducible, and have good interpersonal and intrapersonal reliability [25]. Self-reported pain is the most reliable indicator of pain severity and, if patients are unable to report on pain, pain behavioural tools may be used to estimate pain severity [17, 64]. Although this scoping review identified a limited number of studies, the data show that practitioner behaviour in terms of assessing pain severity may be an area of concern needing further investigation and explanation.

Research which focuses on developing an understanding of the various challenges faced when assessing pain in the African prehospital setting is indicated. In addition, research should aim to determine pain assessment enablers and the development of pain assessment policies/strategies to guide practice and to ensure appropriate education for ECPs to facilitate effective pain assessment in the prehospital setting. Furthermore, to monitor the quality of prehospital acute pain care, EMS systems can incorporate acute pain assessment and management as clinical quality indicators and implement CQI programs to improve the quality of and accountability for prehospital pain care. The study by Scott et al. [45] is indicative of the value CQI programs may have on the delivery of quality prehospital EC and acute pain care.

### 4.4. Prehospital Acute Pain Management

Mulder [40], Matthews et al. [41], and Vincent-Lambert and De Kock [42] reported on the use of morphine and, depending on the level of qualification, the use of ketamine in the prehospital setting. As a result of levels of ECP qualifications restricting the pharmacological scope of practice as well as logistical and cost issues related to inhaled nitrous oxide, many patients treated and transported by EMS in SA may not receive prehospital pain management. Pain management practice in the Rwandan EMS system appears to be unique as prehospital care in the study's cohort was provided by nurses and anaesthesia technicians with a broad array of pain medications (acetaminophen, ibuprofen, diclofenac, morphine, tramadol, fentanyl, pethidine, and ketamine) at their disposal [45]. In the rest of Africa, access to pain management in the prehospital setting would likely be more limited, due to scope of practice confines and other EMS system-related limitations. In SA, some of the limitations in the provision of pain management in the prehospital setting would likely be addressed by the recently revised evidence-based CPGs [44]. Similar pain management frameworks relevant to the African prehospital setting, whether novel or based on international practice, are needed. Research should focus on in-depth investigation and evaluation to develop appropriate policies/strategies for pain management and practitioner education in terms of pain management. Undoubtedly, the development of the CPGs [44] for the South African prehospital setting demonstrates growth in the profession and will prove valuable for quality patient care. Nonetheless, considering the limited resources and the lack of ECPs trained to a level higher than BLS in the rest of Africa, it must be questioned whether the CPGs are adaptable to other EMS systems in Africa.

Included studies provided limited evidence on nonpharmacological pain management, making it a further aspect requiring additional investigation in the African prehospital setting. The literature review by Pak et al. [66] determined that evidence indicates the potential for nonpharmacological pain management choices to play a vital role and likely decrease the use of medications.

### 4.5. Study Limitations

An attempt was made to ensure that all unpublished literature on the topic of the scoping review was accessed by searching grey literature and contacting leaders in EM in Africa. Nevertheless, relevant unpublished articles or thesis dissertations may still have been missed. Despite the extended search, a very limited number of studies could be identified, and as a result, the implications for practice are limited but there are significant implications for research as the review clarifies research gaps and assists in directing focus.

The majority (83%) of the studies included in the scoping review were conducted in the South African prehospital setting; this can most likely be attributed to the immaturity or lack of EMS systems in most African countries [20] as well as be an indication of the limited research capacity in Africa. As a result, the findings of the scoping review are probably a true representation of the paucity of prehospital pain research in Africa. In comparison to other African countries, SA probably possesses the most developed EMS system, employing ECPs with university-level qualifications, thus more likely to perform research.

A further drawback to the findings of the scoping review is that none of the included studies represented data on the patients' perspective of the quality of pain management (satisfaction), but then this may, as well, be attributed to the scarcity and immaturity of research in the African prehospital setting.

Scoping review methodology does not generally require the critical appraisal of the quality of the included studies [34, 37, 38]; consequently, the quality of included studies in the scoping review was not assessed. This issue remains a critique and controversy [35] in the methodology of scoping reviews and therefore deemed a limitation [36] of this scoping review.

## 5. Conclusion

### 5.1. Implications for Research

Acute pain research in the African prehospital setting is significantly lacking, and large knowledge gaps exist. In order to fill the research gaps in the African prehospital setting and develop the profession, it is paramount that research capacity amongst members of the EC profession is built through education and training and that governments invest in the development of EMS systems and quality prehospital care.

In terms of acute pain, it is recommended that research should focus on the following pertinent areas: gathering and publishing epidemiology data related to acute pain in the African prehospital setting, understanding providers' practice as well as barriers to and enablers of pain assessment and management in the African prehospital setting, identifying limitations within EMS systems and limitations in scope of practice, and developing evidence-based CPGs for pain assessment and management catering for all EMS systems in Africa.

### 5.2. Implications for Practice

Due to the limited number of studies included in the scoping review, deducing implications for practice is problematic. Educational initiatives to improve the knowledge and understanding of pain assessment and management principles may prove beneficial to the quality of acute pain care. Additionally, if the scope of practice for each level of ECP qualification includes medication(s) appropriate to alleviate pain yet fitting for the level of qualification, suffering secondary to acute pain will be reduced and patient outcomes improved. Introducing pain assessment and management as EMS quality indicators will allow services to start evaluating pain care and allow for the development of CQI initiatives to advance patient care and outcomes.

## Figures and Tables

**Table 1:** Included source characteristics.

Characteristics	Number (*n*=6)	Percentage (%)
Publication year		
2012	1	16.7
2015	2	33.3
2017	2	33.3
2018	1	16.7
Publication type		
Journal article (peer reviewed)	4	66.6
Thesis dissertation	1	16.7
Grey literature	1	16.7
Countries of origin		
South Africa	5	83.3
Rwanda	1	16.7
Research methods (primary and secondary research)		
Mixed methods (primary research)		
Sequential exploratory	1	16.7
Quasiexperimental (primary research)		
Interrupted time series analysis	1	16.7
Descriptive observational studies (primary research)		
Cross-sectional study	1	16.7
Survey	2	33.3
Secondary research		
Evidence-based clinical practice guideline	1	16.7
Area of intervention (clinical, educational, policy, etc.)		
Clinical	6	100
Language of publication		
English	6	100

**Table 2:** Overview of included studies.

Author(s), year of publication	Study design	Study aim(s)	Study setting	Data collection period	Study sample	Results		
						Pain assessment (initial and reassess)	Nonpharmacological management	Pharmacological management
Mulder, 2012 [40]	Mixed methods: sequential exploratory	To determine the factors contributing to the clinical decision-making process made by South African paramedics in their management of patients with acute traumatic pain	South Africa	Phase 1: quantitative (descriptive cross-sectional study)				
				7 June–30 September 2010	*N*=57 participants (ALS), 22% response rate	*Initial*: analgesia initiated based on a comprehensive clinical picture *Reassess*: both decreased pain score and physiological indicator of change	Positioning and splinting	Morphine, ketamine, voltaren, NSAIDs, tramadol, benzodiazepines
				Phase 2: qualitative (in-depth interviews)				
				2010	*N*=5 participants (ALS)	*Initial*: main determinant in decision-making around initiating analgesia was the patient's expression (verbal) of pain *Reassess*: participants rely on the patient's expression of pain relief rather than a numerical score in the decision-making process	Not reported	Morphine or ketamine (preferred when in scope) or alternatively a combination of morphine and ketamine

Matthews et al., 2017 [41]	Descriptive retrospective survey	To describe prehospital pharmacological analgesia practices in the city of Cape Town	Cape Town, South Africa	August 2013–July 2014	530 PCRs (ALS employees of WCEMS)	*Initial*: NRS assessed in 21% (*n*=111) of PCRs *Reassess*: 2^nd^ NRS assessed in 6% (*n*=34) of PCRs	Not reported	Nitrates administered in 37% (*n*=197), morphine in 75% (*n*=278), and ketamine in 1.7% (*n*=9) of cases

Vincent-Lambert and De Kock, 2015 [42]	Prospective descriptive study: internet-based survey	To describe the use of morphine sulphate and compare paramedic practices to existing guidelines and literature	South Africa	One month in 2015	*N*=60 participants (ALS), 38% response rate	*Initial*: not reported *Reassess*: stop pain management partly based on decreased pain score	Not reported	Morphine

Cox et al., 2015 [43]	Descriptive cross-sectional study	To assess the community management of paediatric burns prior to admission to a burns centre against the current provincial policy guidelines and to identify areas for improvement	Cape Town, South Africa	August–October 2012 and June–August 2013	*N*=353 paediatric burn patients (aged 1 month to 14 years)	*Initial*: not performed *Reassess*: not performed	Cooling with water, ice or cooling agents like Burnshield® Burnshield® applied by EMS in 6.2% (*n*=22) children	Paracetamol, NSAID, tilidine, morphine, and ketamine

HPCSA, 2018 [44]	Adaptive CPG design	To review and update existing protocols for ECPs and create an evidence-based CPG which provides an evidence base for emergency care practice contextualised to the South African setting, is patient-centred, realistic, and enhances continuity of care throughout the emergency system, and is aligned to best practice and provide guidance to current practitioners and those envisioned by the draft NECET policy	South Africa	Searching: October 2015–January 2016	276 CPGs included	*Initial*: assess pain as part of general patient care *Reassess*: use age-appropriate pain assessment scale, reassess every 5 minutes Observe for evidence of serious adverse effects	*Burns*: cool and cover burns *Fracture*: effectively immobilise fracture No further recommendation specifically related to nonpharmacological pain management	*Labour*: inhaled nitrous oxide or opioids (IV or IM) *Chest pain (dependent on the cause)*: sublingual or IV nitrates and/or opioids (IV or IM) *Burns*: paracetamol or NSAIDs, consider opioids for intermittent or procedural pain *Trauma*: narcotic analgesics (morphine IV or fentanyl IV/IN) for moderate to severe pain *Procedural sedation and analgesia*: ketamine: IV, if sedation inadequate, incremental IV doses *Postresuscitation care*: pain and discomfort should be controlled with analgesics and sedatives

Scott et al., 2017 [45]^*∗*^	Quasi-experimental: interrupted time series analysis	To compared five quality process measures recorded before and after the implementation of the CQI programme and aimed to determine the immediate impact of the CQI programme as well as the impact over time	Kigali, Rwanda	Pre-CQI: January 2013–February 2014 Post-CQI: April 2014–May 2015	*N*=1028 trauma patients >15 years	*Initial*: not reported *Reassess*: not reported	Splinting of long bone fractures: Pre-CQI: 87.5% (*n*=335) Post-CQI: 92.6% (*n*=393) *p* value: 0.019	Acetaminophen, ibuprofen, diclofenac, morphine, tramadol, fentanyl, pethidine and ketamine. Pain management for long bone fractures: pre-CQI: 85.1% (*n*=335) Post-CQI: 93.6% (*n*=393) *p* value: <0.001

^*∗*^No formal prehospital care-certified programme was available, and thus, ambulances in Rwanda are manned by one driver, one anaesthesia technician, and one nurse. ALS: advanced life support; CPGs: clinical practice guidelines; CQI: continuous quality improvement; ECPs: emergency care providers; EMS: emergency medical services; IM: intramuscular; IN: intranasal; IV: intravenous; NECET: National Emergency Care Education and Training; NRS: numeric rating scale.

**Table 3:** Summary of pharmacological management of pain as per HPSCA CPGs [44].

Indication	Description
Labour	(i) Inhaled nitrous oxide is the recommended method for pain relief (ii) Practitioners need to explain that medication results in moderate pain relief and ensure that the patient understands possible adverse effects (iii) If IV or IM opioids are considered, inform the patient of limited effect

Trauma (moderate to severe pain)	(i) Morphine (IV) or fentanyl (IV or IN) is recommended (ii) Morphine: IV 0.1 mg/kg or fentanyl: IV/IN 1.0 *µ*g/kg (adult IN dose) (iii) Paediatric IN fentanyl dose: 1.5 *µ*g/kg (iv) If pain remains noteworthy, consider redosing with half the initial dose

Burns	(i) Appropriately manage pain (ii) Administer paracetamol or NSAIDs to manage pain (iii) Opioids can be considered for intermittent pain or pain associated with procedures

Chest pain (management dependent on cause)	(i) Chest pain at first contact: sublingual or IV nitrates while titrating to blood pressure and/or (ii) Opioids titrated and used with caution to limit potential interaction with antiplatelet therapy

Procedural sedation and analgesia	(i) Ketamine IV, IN, or IM is recommended, followed by additional incremental IV doses of ketamine if sedation inadequate (ii) Loading dose over 30–60 sec: adults IV 1 mg/kg and paediatrics IV 1.5–2 mg/kg (iii) If sedation is inadequate or repeated dose necessary, administer additional incremental doses of 0.5–1 mg/kg IV (iv) Alternative to IV administration: IM 4-5 mg/kg or IN (no dose stipulated)

Postresuscitation care	(i) Opioids (morphine or fentanyl) and sedation can be administered to control pain and discomfort

IM: intramuscular; IN: intranasal; NSAIDs: nonsteroidal anti-inflammatory drugs; IV: intravenous.

## Supplementary Materials

Supplementary 1 Appendix 1: PubMed search strategy.

Supplementary 2 Appendix 2: Eligibility form.

Supplementary 3 Appendix 3: Data extraction form.
