# Supplementary material for: Acute Pain in the African Prehospital Setting: A Scoping Review
Source: Pain Res Manag. 2019 Apr 16;2019:2304507. doi: 10.1155/2019/2304507 (PMC6501243; doi:10.1155/2019/2304507)
Supplement: Supplementary 1 — Appendix 1: PubMed search strategy. [file 2304507.f1.pdf]

Appendix 1: **PubMed Search Strategy**

#1 Developing Countries[Mesh:noexp] OR Africa[Mesh:noexp] OR Africa, Northern[Mesh:noexp] OR Africa South of the Sahara[Mesh:noexp] OR Africa, Central[Mesh:noexp] OR Africa, Eastern[Mesh:noexp] OR Africa, Southern[Mesh:noexp] OR Africa, Western[Mesh:noexp] OR Angola[Mesh:noexp] OR Benin[Mesh:noexp] OR Botswana[Mesh:noexp] OR Burkina Faso[Mesh:noexp] OR Burundi[Mesh:noexp] OR Cameroon[Mesh:noexp] OR Cape Verde[Mesh:noexp] OR Central African Republic[Mesh:noexp] OR Chad[Mesh:noexp] OR Comoros[Mesh:noexp] OR Congo[Mesh:noexp] OR Cote d'Ivoire[Mesh:noexp] OR Djibouti[Mesh:noexp] OR "Democratic Republic of the Congo"[Mesh:noexp] OR Eritrea[Mesh:noexp] OR Ethiopia[Mesh:noexp] OR Gabon[Mesh:noexp] OR Gambia[Mesh:noexp] OR Ghana[Mesh:noexp] OR Guinea[Mesh:noexp] OR Guinea-Bissau[Mesh:noexp] OR Kenya[Mesh:noexp] OR Lesotho[Mesh:noexp] OR Liberia[Mesh:noexp] OR Libya[Mesh:noexp] OR Madagascar[Mesh:noexp] OR Malawi[Mesh:noexp] OR Mali[Mesh:noexp] Mauritania[Mesh:noexp] OR Mauritius[Mesh:noexp] OR Mozambique[Mesh:noexp] OR Namibia[Mesh:noexp] OR Niger[Mesh:noexp] OR Nigeria[Mesh:noexp] OR Rwanda[Mesh:noexp] OR Senegal[Mesh:noexp] OR Seychelles[Mesh:noexp] OR Sierra Leone[Mesh:noexp] OR Somalia[Mesh:noexp] OR South Africa[Mesh:noexp] OR Sudan[Mesh:noexp] OR Swaziland[Mesh:noexp] OR Tanzania[Mesh:noexp] OR Togo[Mesh:noexp] OR Uganda[Mesh:noexp] OR Zambia[Mesh:noexp] OR Zimbabwe[Mesh:noexp]

#2 Madagascar[tw] OR Malagasy Republic[tw] OR Malawi[tw] OR Nyasaland[tw] OR Mali[tw] OR Mauritania[tw] OR Mauritius[tw] OR Agalega Islands[tw] OR Mozambique[tw] OR Namibia[tw] OR Niger[tw] OR Nigeria[tw] OR Rwanda[tw] OR Ruanda[tw] Sao Tome[tw] OR Senegal[tw] OR Seychelles[tw] OR Sierra Leone[tw] OR Somalia[tw] OR Sudan[tw] OR Swaziland[tw] OR Tanzania[tw]OR Togo[tw] OR Togolese Republic[tw] OR Uganda[tw] OR Zambia[tw] OR Zimbabwe[tw] OR Rhodesia[tw]

#3 Africa[tw] OR Angola[tw] OR Benin[tw] OR Botswana[tw] OR Burkina Faso[tw] OR Burkina Fasso[tw] OR Upper Volta[tw] OR Burundi[tw] OR Urundi[tw] OR Cameroon[tw] OR Cameroons[tw] OR Cameron[tw] OR Camerons[tw] OR Cape Verde[tw] OR Central African Republic[tw] OR Chad[tw] OR Comoros[tw] OR Comoro Islands[tw] OR Comores[tw] OR Mayotte[tw] OR Congo[tw] OR Zaire[tw] OR Cote d'Ivoire[tw] OR Ivory Coast[tw] OR Djibouti[tw] OR French Somaliland[tw] OR Ethiopia[tw] OR Gabon[tw] OR Gabonese Republic[tw] OR Gambia[tw] OR Ghana[tw] OR Gold Coast[tw] OR Guinea[tw] OR Guiana[tw] OR Kenya[tw] OR Lesotho[tw] OR Basutoland[tw] OR Liberia[tw] OR Libya[tw]

#4 "developing country"[tw] OR "developing countries"[tw] OR "developing nation"[tw] OR "developing nations"[tw] OR "developing population"[tw] OR "developing populations"[tw] OR "developing world"[tw] OR "less developed country"[tw] OR "less developed countries"[tw] OR "less developed nation"[tw] OR "less developed nations"[tw] OR "less developed population"[tw] OR "less developed populations"[tw] OR "less developed world"[tw] OR "lesser developed country"[tw] OR "lesser developed countries"[tw] OR "lesser developed nation"[tw] OR "lesser developed nations"[tw] OR "lesser developed population"[tw] OR "lesser developed populations"[tw] OR "lesser developed world"[tw] OR "under developed country"[tw] OR "under developed countries"[tw] OR "under developed nation"[tw] OR "under developed nations"[tw] OR "under developed population"[tw] OR "under developed populations"[tw] OR "under developed world"[tw] OR "underdeveloped country"[tw] OR "underdeveloped countries"[tw] OR "underdeveloped nation"[tw] OR "underdeveloped nations"[tw] OR "underdeveloped population"[tw] OR "underdeveloped populations"[tw] OR "underdeveloped world"[tw] OR "middle income country"[tw] OR "middle income

countries"[tw] OR "middle income nation"[tw] OR "middle income nations"[tw] OR "middle income population"[tw] OR "middle income populations"[tw] OR "low income country"[tw] OR "low income countries"[tw] OR "low income nation"[tw] OR "low income nations"[tw] OR "low income population"[tw] OR "low income populations"[tw] OR "lower income country"[tw] OR "lower income countries"[tw] OR "lower income nation"[tw] OR "lower income nations"[tw] OR "lower income population"[tw] OR "lower income populations"[tw] OR "underserved country"[tw] OR "underserved countries"[tw] OR "underserved nation"[tw] OR "underserved nations"[tw] OR "underserved population"[tw] OR "underserved populations"[tw] OR "underserved world"[tw] OR "under served country"[tw] OR "under served countries"[tw] OR "under served nation"[tw] OR "under served nations"[tw] OR "under served population"[tw] OR "under served populations"[tw] OR "under served world"[tw] OR "deprived country"[tw] OR "deprived countries"[tw] OR "deprived nation"[tw] OR "deprived nations"[tw] OR "deprived population"[tw] OR "deprived populations"[tw] OR "deprived world"[tw] OR "poor country"[tw] OR "poor countries"[tw] OR "poor nation"[tw] OR "poor nations"[tw] OR "poor population"[tw] OR "poor populations"[tw] OR "poor world"[tw] OR "poorer country"[tw] OR "poorer countries"[tw] OR "poorer nation"[tw] OR "poorer nations"[tw] OR "poorer population"[tw] OR "poorer populations"[tw] OR "poorer world"[tw] OR "developing economy"[tw] OR "developing economies"[tw] OR "less developed economy"[tw] OR "less developed economies"[tw] OR "lesser developed economy"[tw] OR "lesser developed economies"[tw] OR "under developed economy"[tw] OR "under developed economies"[tw] OR "underdeveloped economy"[tw] OR "underdeveloped economies"[tw] OR "middle income economy"[tw] OR "middle income economies"[tw] OR "low income economy"[tw] OR "low income economies"[tw] OR "lower income economy"[tw] OR "lower income economies"[tw] OR "low gdp"[tw] OR "low gnp"[tw] OR "low gross domestic"[tw] OR "low gross national"[tw] OR "lower gdp"[tw] OR "lower gnp"[tw] OR "lower gross domestic"[tw] OR "lower gross national"[tw] OR lmic[tw] OR “lmics"[tw] OR "third world"[tw] OR "lami country"[tw] OR "lami countries"[tw] OR "transitional country"[tw] OR "transitional countries"[tw]
#5 #1 OR #2 OR #3 OR #4
#6 “Emergency Medical Services"[Mesh] OR "Emergency Medicine"[Mesh] OR "Emergency Treatment"[Mesh] OR emergenc* OR prehospital* OR pre-hospital* OR out-of-hospital OR “out of hospital” Or “before hospital” OR “prior to hospital” OR ambulance* OR "Ambulances"[Mesh] OR paramedic* OR "Emergency Medical Technicians"[Mesh] OR "Emergency Responders"[Majr]
#7 "Acute Pain"[Mesh] OR "Pain"[Mesh] OR pain OR "Analgesia"[Mesh] OR analgesia OR "Pain Management"[Mesh]
#8 #5 AND #6 AND #7
#9 #8 AND Filters: Publication date from 2000/01/01
