# Supplementary material for: Acute Pain in the African Prehospital Setting: A Scoping Review
Source: Pain Res Manag. 2019 Apr 16;2019:2304507. doi: 10.1155/2019/2304507 (PMC6501243; doi:10.1155/2019/2304507)
Supplement: Supplementary 2 — Appendix 2: Eligibility form. [file 2304507.f2.pdf]

Acute Pain in the African Pre-hospital Setting: A Scoping Review

Scoping Review Eligibility Form:

Scoping Review Aim:
The overall aim of this review is to identify and map the body of evidence related to acute pain assessment and management in the pre-hospital setting, in Africa and to identify gaps in current evidence.

Reviewer Details:

**Reviewer:**	**Date Assessed:**
Choose an item.	

Study Details:

**Authors:**	**Year Published:**
**Study Title:**	**Reference Number:**

1) Type of Research:

**Question:**		
**Do the study design include a design as stipulate (inclusion criteria) in the list below?**<br>• Review research designs: Systematic reviews and meta-analysis<br>• Experimental designs: Randomized Controlled Trials (RCT) or Non-randomized controlled trails or quasi-experimental<br>• Observational studies: Cohort studies, Case-control studies, Cross-sectional studies or Surveys<br>• Qualitative design<br>• Evidence-based guidelines<br><br>**Note:** The following study designs will not be included: Literature reviews, Case Reports and Case Series	Yes	☐
	No	☐
	Unclear	☐

2) Type of Participants:

**Question:**		
**Do the study population include adults and paediatrics (> 28 days or 1 month) participants?**<br>**OR**<br>**Pain assessment and management by emergency care providers, physicians and/or nurses in the pre-hospital setting.**<br>**Note:** Neonates (0-28 days) will not be included.	Yes	☐
	No	☐
	Unclear	☐

3) Timeframe:

**Question:**		
**Was the study conducted on or after the 1st of January 2000?**<br><br>**Note:** Studies conducted before the 1st of January 2000 will not be included.	Yes	☐
	No	☐
	Unclear	☐

4) Study Setting:

**Question:**		
**Is the study conducted in the pre-hospital setting in Africa [studies conducted in the aero-medical (helicopter and fixed-wing) setting and ground ambulance services will be included]?**<br><br>**Note:** Studies conducted in-hospital and studies related to inter-facility transfers of critically ill and injured patients will not be included.	Yes	☐
	No	☐
	Unclear	☐

5) Eligible for Inclusion:

**Question:**		
**Do the study meet all the inclusion criteria?**	Yes	☐
	No	☐
	Unclear	☐

6) Comments (stipulate any comments, concerns or uncertainty regarding eligibility):

