# Supplementary material for: Acute Pain in the African Prehospital Setting: A Scoping Review
Source: Pain Res Manag. 2019 Apr 16;2019:2304507. doi: 10.1155/2019/2304507 (PMC6501243; doi:10.1155/2019/2304507)
Supplement: Supplementary 3 — Appendix 3: Data extraction form. [file 2304507.f3.pdf]

Acute Pain in the African Pre-hospital Setting: A Scoping Review

Scoping Review Data Extraction Form

Reviewer Details:

**Reviewer:**	**Date Assessed:**
Choose an item.	

Study Details:

**Authors:**	**Year Published:**
**Study Title:**	**Reference Number:**

1) Research aims:

   Click or tap here to enter text.

2) Study Location and Study Setting (if information available):
   - Click or tap here to enter text.
   - Click or tap here to enter text.
   - Click or tap here to enter text.
   - Click or tap here to enter text.

3) Year of publication and study period:
   - Click or tap here to enter text.
   - Click or tap here to enter text.

4) Publication type (journal article, dissertation, conference processing, etc.)
   - Click or tap here to enter text.
   - Click or tap here to enter text.
   - Click or tap here to enter text.

Methodology:

5) Type of Study Design:
   - Choose an item.
   - Click or tap here to enter text.

6) Data collection method (interviews, questionnaires, patient care report reviews, etc.)
   - Click or tap here to enter text.
   - Click or tap here to enter text.

7) Sampling Strategy and Sample size:
   - Click or tap here to enter text.
   - Click or tap here to enter text.

8) Data Collection:

Click or tap here to enter text.

9) Data analysis and measures:

Click or tap here to enter text.

Participants:

10) Participant or Practitioner level of qualification/s:

- Click or tap here to enter text.

11) Type of participant:

- Medical or Trauma: Choose an item.
- Click or tap here to enter text.
- Patient type: Choose an item.
- Click or tap here to enter text.

If applicable:

12) Type of pain assessment tool or tools

1. Click or tap here to enter text.

   Click or tap here to enter text.
2. Click or tap here to enter text.

   Click or tap here to enter text.
3. Click or tap here to enter text.

   Click or tap here to enter text.

13) Medication administered to patients:

- Choose an item.

14) Medication information (provide if Question 10 was answered Yes'):

- Class of medication/s: Click or tap here to enter text.
- Medication/s administered: Click or tap here to enter text.
- Dose administered: Click or tap here to enter text.
- Repeated dosages: Click or tap here to enter text.
- Rescue analgesia: Click or tap here to enter text.
- Click or tap here to enter text.

15) Non-Pharmacological Management of Pain:

- Click or tap here to enter text.

16) Route of administration for each medication (inhaled, oral, intranasal, intramuscular, intravenous)

- Click or tap here to enter text.

Key findings related to the Scoping Reviews aims and objectives:

**Aim:**	The overall aim of this review is to identify and map the body of evidence related to acute pain assessment and management in the pre-hospital setting, in Africa and to identify gaps in current evidence.
**Objectives:**	• To identify and map the range and nature of evidence in relation to acute pain assessment and management in the pre-hospital setting, in Africa.<br>• To identify research gaps in the existing literature related to acute pain assessment in the pre-hospital setting, in Africa.<br>• To summarize research findings related to acute pain assessment and management in the pre-hospital setting, in Africa.<br>• To inform future research related to acute pain assessment in the pre-hospital setting, in Africa.

17) Results (Quantitative data):

No:	Primary Outcome:	Details:
1	Click or tap here to enter text.	Click or tap here to enter text.
2	Click or tap here to enter text.	Click or tap here to enter text.
3	Click or tap here to enter text.	Click or tap here to enter text.
4	Click or tap here to enter text.	Click or tap here to enter text.
5	Click or tap here to enter text.	Click or tap here to enter text.
6	Click or tap here to enter text.	Click or tap here to enter text.
7	Click or tap here to enter text.	Click or tap here to enter text.
8	Click or tap here to enter text.	Click or tap here to enter text.
9	Click or tap here to enter text.	Click or tap here to enter text.
10	Click or tap here to enter text.	Click or tap here to enter text.
11	Click or tap here to enter text.	Click or tap here to enter text.
12	Click or tap here to enter text.	Click or tap here to enter text.

18) Results (Qualitative data):

No:	Themes:	Description:
**1**	Click or tap here to enter text.	Click or tap here to enter text.
**2**	Click or tap here to enter text.	Click or tap here to enter text.
**3**	Click or tap here to enter text.	Click or tap here to enter text.
**4**	Click or tap here to enter text.	Click or tap here to enter text.
**5**	Click or tap here to enter text.	Click or tap here to enter text.
**6**	Click or tap here to enter text.	Click or tap here to enter text.
**7**	Click or tap here to enter text.	Click or tap here to enter text.
**8**	Click or tap here to enter text.	Click or tap here to enter text.

19) Other information:

Click or tap here to enter text.

Click or tap here to enter text.

Click or tap here to enter text.

Click or tap here to enter text.

Click or tap here to enter text.

20) Comments (stipulate any comments, concerns or uncertainty regarding eligibility):

Click or tap here to enter text.
